# Medical Department of the University of Buffalo

**Published:** 1855-08

**Authors:** 


*Medical Department of the University of Buffalo.*—The Annual Announcement of this school is out, and now in process of circulation. The Faculty remains as heretofore organized. The preliminary term will commence as usual at the beginning of October, and the regular lecture course a month later.

We will be pardoned if we call the attention of our readers to the advantages offered by Buffalo for the purpose of medical teaching. Whatever may be said by interested parties to the contrary, no one doubts that good clinical opportunities form one of the great essentials of a medical school. To secure these a large tributary population is necessary. The census now nearly completed shows that Buffalo will fall but little, if any, short of 100,000 inhabitants. Buffalo has but one hospital, and that is a large and well conducted institution, on the same block with the college, and open to all the students of the school, immediately on paying their matriculation ticket, no extra fee being required. Two half days in each week are devoted to clinical lectures in the commodious theatre provided in the Hospital for the purpose, or at the bedside of the patient. All the more important surgical operations pass under the eye of the student, and, to ensure the fullest benefit from this form of teaching, cases are assigned to students, upon which they are required to give a written diagnosis, prognosis, and treatment, to be handed in as a thesis to the professor of practice on surgery. Careful study of individual cases is thus secured, and the student familiarized with medical composition preparatory to his inaugural thesis. In the department of obstetrics, unusual opportunities are offered to the graduating class, who are admitted to the lying-in room, both before and during labor, taught the sounds of the fœtal heart, and fully accustomed to the manipulations and etiquette of obstetricy.

Important improvements in the comfort of the building will be made during the summer, to remedy some defects in a building otherwise unexcelled in beauty and convenience.

We hope and expect to chronicle the presence of a large class at the opening of the lecture season, and the existence at its close of the same harmony and good feeling, which have heretofore subsisted between teacher and pupil at this institution.

