# Correction

**Published:** 1855-09

**Authors:** 


*Correction.*—A curious perversion of sense and truth, by the error of a single letter, occurs in our last. In speaking of the means of clinical instruction afforded at the University of Buffalo, we described a method—first proposed and carried out by Prof. Rochester—in which to quote ourselves: "cases are assigned, upon which they (students) are required to give a written prognosis, diagnosis, and treatment, to be handed in a thesis to the professor of practice *on* surgery." The word "on" should, of course, read "or," this change of a single letter restoring sense to an otherwise senseless sentence.

